# Assessing the environmental characteristics of cycling routes to school: a study on the reliability and validity of a Google Street View-based audit

**DOI:** 10.1186/1476-072X-13-19

**Published:** 2014-06-10

**Authors:** Griet Vanwolleghem, Delfien Van Dyck, Fabian Ducheyne, Ilse De Bourdeaudhuij, Greet Cardon

**Affiliations:** 1 Department of Movement and Sport Sciences, Faculty of Medicine and Health Sciences, Ghent University, Watersportlaan 2, 9000 Ghent, Belgium; 2 Research Foundation Flanders (FWO), Egmontstraat 5, 1000 Brussels, Belgium

**Keywords:** Google Street View, Active commuting to school, Cycling routes, Children, Physical environment

## Abstract

**Background:**

Google Street View provides a valuable and efficient alternative to observe the physical environment compared to on-site fieldwork. However, studies on the use, reliability and validity of Google Street View in a cycling-to-school context are lacking. We aimed to study the intra-, inter-rater reliability and criterion validity of EGA-Cycling (*E*nvironmental *G*oogle Street View Based *A*udit - Cycling to school), a newly developed audit using Google Street View to assess the physical environment along cycling routes to school.

**Methods:**

Parents (n = 52) of 11-to-12-year old Flemish children, who mostly cycled to school, completed a questionnaire and identified their child’s cycling route to school on a street map. Fifty cycling routes of 11-to-12-year olds were identified and physical environmental characteristics along the identified routes were rated with EGA-Cycling (5 subscales; 37 items), based on Google Street View. To assess reliability, two researchers performed the audit. Criterion validity of the audit was examined by comparing the ratings based on Google Street View with ratings through on-site assessments.

**Results:**

Intra-rater reliability was high (kappa range 0.47-1.00). Large variations in the inter-rater reliability (kappa range -0.03-1.00) and criterion validity scores (kappa range -0.06-1.00) were reported, with acceptable inter-rater reliability values for 43% of all items and acceptable criterion validity for 54% of all items.

**Conclusions:**

EGA-Cycling can be used to assess physical environmental characteristics along cycling routes to school. However, to assess the micro-environment specifically related to cycling, on-site assessments have to be added.

## Background

Physical activity for children provides numerous health benefits on both physical [1] and mental level [2]. Engaging in walking and cycling to school represents an important proportion of the daily physical activity in 6-to-12-year olds [3-6]. In some European countries, like in Belgium (Flanders), cycling to school is more common than walking to school among 10-to-13-year old children [7,8]. An important advantage of cycling is the possibility to travel considerable distances at higher speeds. Additionally, from the age of 11, children create a higher level of independent mobility and cycle to school or to other destinations independently [5,9]. So focusing on cycling behavior in this age group (11-to-12 years) is important. Despite the fact that cycling to school is an established common behavior in Flanders, 47.7% of all 11-to-12-year old passive commuters is living within a feasible cycling distance (three kilometers) to school [8]. Therefore, the focus of this study is on cycling to school among 11-to-12-year old children.

To get insight into the determinants of cycling to school, socio-ecological models identify correlates at multiple levels (individual, social and physical environmental factors) [10,11]. Specifically, there is growing interest in examining the relationship between the physical environment and active transportation to school in elementary school children [4,5,12], but few studies reported specific results for cycling to school [13,14]. The few studies that have been conducted until now, identified several physical environmental factors as predictors of children’s cycling to school: number of recreation facilities, presence of green space, frequency of sidewalks, traffic lights [14] and cycling facilities around the home or school environment [13]. However, accurately assessing the physical environment in a cycling context remains challenging.

Up to now, the physical environment has mainly been assessed by self-reported questionnaires [15,16], with bias and conflicting results as frequently reported problems [17,18]. Therefore, observational field audits are commonly used to objectively study the environmental factors related to physical activity [19,20]. Audits are frequently conducted to obtain an objective rating of the physical environment and can give objective information on a micro-level. Frequently used audit tools, such as Pikora-SPACES [21], Irvine-Minnesota Inventory [22], Audit Tool Checklist and Analytic Version [23] and PEDS [19] assess detailed information such as the presence, quality, continuity and inclination gradient of walking and cycling facilities. Traffic and personal safety measures are also included, based on the presence or absence of crossings, crossing aids, lighting, surveillance etc. The aesthetics of the environment can also be assessed with audit tools, in order to obtain information about the overall attractiveness of the physical environment. However, conducting audits is resource- and time-intensive because researchers have to travel to the specific location to observe the environment [24]. Currently, there is a growing research interest in using new technologies with high-resolution omnidirectional images to provide a visual assessment of the environmental setting. For example, Google Street View has been applied to objectively assess the physical environment in physical activity research [25-33]. Its omnidirectional camera systems allow the user to virtually walk through the streets and observe the environment as in real-time. A study of Badland and colleagues [26] showed that conducting the virtual audit by Google Street View was much quicker than field assessments. Furthermore, a study of Kelly and colleagues [32] showed good inter-rater reliability (95% of the items had substantial to nearly perfect agreement). Additionally, recent studies have shown that observations of the neighborhood environment conducted by Google Street View have a good validity against field audits [26,27,29]. However, the level of agreement between a virtual neighborhood audit instrument using Google Street View and in-person field work was lower when qualitative and more detailed data (e.g. quality of street conditions, presence of garbage) and temporally items (e.g. traffic volume) were assessed [27,29].

Previous studies reported reliability and validity of Google Street View-based audits for observations of neighborhood environments. However, for a cycling-to-school context, additional environmental factors along the routes (e.g. swerving alternatives for cyclists, separate cycle lanes not allowing car traffic) are important. They may be relevant to get parental allowance to cycle to school or they may influence the child’s preference of travel mode [14,34,35]. Previous studies reported the importance of street design factors (crossings, sidewalks, street connectivity, …) along the route to school, yet they only considered the shortest route taken to school. Wong and colleagues [36] however concluded that environmental characteristics along the shortest route to school may not exactly reflect the environmental characteristics along a child’s actual route to school. Additionally, when cycling along the routes, a different perspective is obtained compared to walking or being in a car. So previously studied Google Street View-based audits may need adaptation for assessing environmental characteristics along a cycling route to school. In addition, no studies used Google Street View in a cycling-to-school context. Reliability and validity of a Google Street View-based audit instrument to assess environmental characteristics along a cycling route to school are lacking. Therefore, the first aim of the present study is to examine the intra- and inter-rater reliability of a newly developed audit instrument (EGA-Cycling (*E*nvironmental *G*oogle Street View Based *A*udit - Cycling to school)) using Google Street View to virtually assess physical environmental characteristics along cycling routes to school among 11-to-12-year old Flemish children. Secondly, the criterion validity of EGA-Cycling is studied using Google Street View against on-site observations filling out the audit.

## Methods

### EGA-cycling

EGA-Cycling [Additional file 1] was developed to assess the physical environmental characteristics of cycling routes to school, using Google Street View. Within the environmental level of the socio-ecological model, the selection of the items of EGA-Cycling was based on their relevance to cycling for transportation [11]. Items specifically associated with children’s cycling behavior were selected to be included in the audit [13,14,34,35,37-46]. The outline of EGA-Cycling and the relevance of the individual items to children’s cycling behavior are presented in an additional file [see Additional file 1]. EGA-Cycling consisted of three main sections ((1) land use, (2) characteristics of the street segment and (3) aesthetics) and included 37 items in total. Eight items were used to assess land use in the corresponding street segments. Questions regarding the mix of residential and non-residential land use (commercial, public and recreational destinations, heavy industry and natural phenomena) were included in this section. A second part of EGA-Cycling included general characteristics of the street segment (12 items; e.g. road type, measures that slow down traffic), cycling facilities (7 items; e.g. type and maintenance cycle lane) and pedestrian facilities (3 items; e.g. maintenance sidewalk). The last part of EGA-Cycling dealt with questions concerning the aesthetics of the street segment (7 items; e.g. presence and maintenance front yards).

EGA-Cycling was based on existing audit instruments (Pikora-SPACES instrument [21], Audit Tool Checklist Version [23], Irvine-Minnesota Inventory [22]). Inter-rater reliability of previous audits was found to be high [21,23,47]. The Audit Tool Checklist Version was primarily used to develop items of EGA-Cycling regarding land use and street characteristics. Some answering options were modified, for instance answering options regarding street infrastructure were adapted to the Flemish street infrastructure (e.g. type of cycle lane) as the Audit Tool Checklist Version was designed for a U.S. environment. More detailed items regarding street characteristics were added and based on the Irvine-Minnesota Inventory as this audit covered more detailed features. The items regarding pedestrian and cycling facilities were partly taken from the SPACES- instrument and partly from the Irvine-Minnesota Inventory with some modifications in answering options. Specific items considered to be relevant to cycling were added to the tool (e.g. swerving alternatives for cyclists, width of cycle lane), based on a report regarding cycling accidents and infrastructure in Flanders showing for instance that a small cycle lane can be a risk for cycling accidents [37]. To observe the aesthetics of the street segment, corresponding items from the 3 existing audits were fitted to the Flemish environment.

### Participants and procedure

Fourteen elementary schools in Flanders were contacted by phone to participate in the present study. The schools were randomly selected out of a list with all elementary schools located in West- and East-Flanders (northern part of Belgium). Finally, 6 elementary schools in Flanders gave permission to participate (two schools in West-Flanders and four in East-Flanders), of which 5 were located in an urban area and one in a suburban area. All parents of the 6^th^ graders (11-to-12-year-old children) were invited to participate in the study that was conducted in the fall of 2012 (November – December). Parents were asked to complete a questionnaire. If parents did not want to participate, they returned the questionnaire unanswered. Questionnaires were distributed and collected through the schools. Parents of 168 sixth graders received a questionnaire and in total 109 parents completed the questionnaire (64.9%). If the children mostly cycled to school, parents had to identify their child’s cycling route to school on an attached street map. The present study was approved by the Ghent University Ethics Committee (EC UZG 2010/246).

Based on the criterion distance of 3 km for cycling from home to school among Flemish school-aged children [8] and taking into account the clarity of the street plan on a A4-size page, a radius between 2 and 3 kilometers around the school was covered on the street map to identify a child’s cycling route. Fifty cycling routes were received (cycling route clearly marked on the street map) of the 52 children whose parents reported they mostly cycled to school (96.2%).

EGA-Cycling was filled out by two researchers to obtain environmental characteristics along a child’s cycling route to school. The selection of two researchers was based on the methodology of similar studies testing reliability and validity of virtual audit tools to assess the physical environment [27,32]. Each cycling route was divided into several street segments (Per route: Mean = 4.5; SD = 2.1) and environmental characteristics in each street segment were audited. Overall, 151 segments were scored (Mean street segment length = 584.3 m; SD = 297.9, range: 389–1900 m). A street segment was defined as the segment between two adjacent intersections or between an intersection and cul-de-sac (dead end) [48]. Segments less than 400 meters were combined with adjacent segments to achieve a distance of at least about 400 meters for the combined segment [48]. Using street segments to assess the physical environment was based on the methodology of prior audits tools (Pikora-SPACES instrument [21], Audit Tool Checklist Version [23] and Irvine-Minnesota Inventory [22]). For each cycling route, all segments were scored. Because EGA-Cycling aims to assess environmental characteristics along children’s entire cycling routes, and not to assess the characteristics in the individual segments, one total score per cycling route was calculated for each item. For each item, the total score consisted of the most often reported answer for all individual segments. To control for differences in length of the segments, the scores of the individual segments were weighted by the distance of the individual segments. The contribution of the reported answers in all street segments on the total score was proportional to the distance of the segment. For each item, the distances of segments with the same reported answer were summed and compared to the sum of the distances of the other answering options. The corresponding answer of the largest sum was the final answer for the entire cycle route.

To assess intra- and inter-rater reliability of EGA-Cycling, 30 cycling routes (15 in urban areas/15 in suburban areas) were randomly selected out of the 50 cycling routes. The selected cycling routes were rated, using Google Street View, by two researchers and both researchers rated the cycling routes twice (period of minimum 1 and maximum 2 weeks between both ratings).

The criterion validity of EGA-Cycling was assessed on 50 cycling routes by comparing ratings from two researchers (same raters as for reliability) using the audit tool in Google Street View with ratings through on-site assessments. A third researcher went to the specific location and cycled along the corresponding cycling routes to rate the environmental characteristics using EGA-Cycling. In order to avoid training effect, the researcher that rated the cycling routes by on-site assessment, did not rate the routes by Google Street View.

### Sociodemographic and active commuting information

Sociodemographic information was obtained through a parental questionnaire. The first section of the questionnaire contained general information about the child (e.g. age, gender). Secondly, parents were asked about their child’s mode of transportation to school using a question matrix [49]. In this matrix parents filled out per season how many days per week their child went to school using different transportation modes ((1) walking, (2) cycling, (3) driven by car and (4) using the public transport).

### Data analysis

The Statistical Package for the Social Sciences for Windows version 20 (SPSS Inc., Chicago, IL, USA) was used to perform statistical analyses, and tests were considered significant at *p* < 0.05. Means, standard deviations (SD) and percentages were used to describe the sample.

#### Intra- and inter-rater reliability

Intra- and inter-rater reliability of EGA-Cycling were assessed by using the kappa test for agreement. To interpret the kappa values, ratings by Landis en Koch [50]: 0.00-0.20 (poor), 0.21-0.40 (fair), 0.41-0.60 (moderate), 0.61-0.80 (substantial), 0.81-1.00 (almost perfect) were used. For the kappa statistics, ratings with negative kappas between -0.10 and 0.00 were interpreted as no agreement since a negative kappa represented agreement worse than expected or disagreement. If no kappas could be calculated, at least one variable was constant indicating no variance in responses of one or both raters. Additionally, percentage agreement was calculated for all items to determine the proportion of occasions that raters gave the same score. Percentage agreement above 70% was considered high [51].

#### *Criterion validity*

Kappa statistics and percentage agreement were calculated between the virtually assessed items and on-site assessments. To interpret the kappa statistics, the same ratings by Landis en Koch were used as for the intra- and inter-reliability. Percentage agreement above 70% was considered high [51].

## Results

### *Description of study sample*

The descriptive characteristics of the total sample (n = 109) are reported in Table 1. Of the 50 children with a valid cycling route to school, 40% (n = 20) were boys and 60% (n = 30) girls. Fifty-four percent of those (n = 27) lived in a suburban area, the other 46% (n = 23) lived in an urban area. Mean age was 11.8 ± 0.8 years.

**Table 1:** Descriptive characteristics of the total sample (n = 109)

	**All**	**Gender**		**Living area**	
		**Boys**	**Girls**	**Suburban**	**Urban**
n (%)	109	52 (47.7)	57 (52.3)	36 (33.0)	73 (67.0)
Age (years) (Mean ± SD)	11.7 ± 0.7	11.7 ± 0.5	11.7 ± 0.8	11.7 ± 0.5	11.7 ± 0.8
Transport mode to school (n,%)					
*Walking*	17 (15.7)	9 (17.6)	8 (14.0)	1 (2.8)	16 (22.2)
*Cycling*	52 (48.1)	24 (47.1)	28 (49.1)	26 (72.2)	26 (36.1)
*Driven by car*	34 (31.5)	14 (27.5)	20 (35.1)	9 (25.0)	25 (34.7)
*Public transport*	5 (4.6)	4 (7.8)	1 (1.8)	0 (0.0)	5 (6.9)

The response frequency of each item on the first assessment of EGA-Cycling audited by rater 1, rater 2 and by on-site rating is shown in an Additional file [see Additional file 2].

### *Intra-rater reliability*

Intra-rater reliability results of EGA-Cycling are presented in an Additional file [see Additional file 3]. Kappa values of the 37 individual items ranged from 0.47 to 1.00, indicating a moderate-to-perfect agreement. In detail, the intra-rater reliability values of 28 individual items were almost perfect, 1 item generated substantial agreement and intra-rater reliability for 2 items was moderate. Moderate agreement was found for the mix of residential/non-residential land use (subscale land use) and the presence of buildings with windows on the street side (subscale general characteristics). No items generated fair or poor intra-rater reliability, kappas could not be calculated for 6 items. Percentage agreement for all individual items ranged from 80% to 100%, indicating a high agreement.

### *Inter-rater reliability*

Results of the inter-rater reliability of EGA-Cycling [see Additional file 3] showed kappas of the 37 individual items ranging from -0.03 to 1.00, indicating no-to-perfect agreement. In total, 16 items generated moderate-to-almost perfect agreement, 8 items fair and 5 items poor or no agreement. Kappas could not be calculated for 8 items. Of the poor scores, one item was categorized under the subscale land use (e.g. “openness of the view”) and the remaining items were categorized under general characteristics. Percentage agreement for all individual items ranged from 36.7% to 100%.

When examining the results by subscale, inter-rater reliability was moderate-to-almost perfect for the subscale land use except for 2 items generating fair (“presence of residential and non-residential land use” (k = 0.37)) and respectively poor agreement (“openness of the view” (k = 0.16)).

In the subscale general characteristics, four items had poor or disagreement: measures that can slow down traffic (k = 0.18), swerving alternatives for cyclists (k = -0.03), presence of driveways (k = 0.17) and garage doors (k = 0.19). For the remaining items in the category general characteristics moderate-to-almost perfect agreement was found with highest scores for posted speed limit (k = 0.83) and measures that make it easier for pedestrians/cyclists to cross over (k = 0.83).

Regarding the category cycling facilities, all items generated fair agreement (e.g. “type of cycle lane” (k = 0.34), “width of cycle lane” (k = 0.23), “two-way cycle lane” (k = 0.32), “maintenance of cycle lane” (k = 0.27), “lighting of cycle lane” (k = 0.27)), except the surface of the cycle lane (k = 0.47) that generated moderate agreement. Concerning pedestrian facilities, all items showed almost perfect agreement.

For the subscale aesthetics, only the items presence of trees and attractive natural features demonstrated moderate agreement, the remaining items showed fair agreement. Lowest scores were found for the items regarding the presence of front yards (k = 0.25) and maintenance of front yards (k = 0.25).

### *Criterion validity*

An additional file [see Additional file 3] shows kappas between the virtually assessed and on-site assessed items.

Kappa values of the 37 items ranged from -0.06 to 1.00, indicating no-to-perfect validity. For 20 of the 37 items the agreement was moderate-to-almost perfect, 7 items generated fair and 6 items poor or no agreement. Of these 6 items, 2 items were classified under the subscale land use, 2 in general characteristics, 1 in cycling facilities and 1 was classified under the subscale aesthetics. Kappas could not be calculated for 4 items. Percentage agreement for all 37 items ranged from 30% to 100%.

In the subscale land use, the majority of the items (6 items) showed a moderate-to-perfect agreement with the highest scores found for the items regarding heavy industry (k = 1.00) and public destinations (k = 0.88). No agreement was found for recreational destinations (k = -0.06) and openness of the view (k = 0.00).

Regarding the general characteristics, more items showed moderate-to-substantial agreement compared to fair-to-poor agreement. The highest score was found for measures that make it easier to cross the street (k = 0.80), lowest scores were found for the items regarding streetlights of the street segment (k = -0.03) and swerving alternatives for cyclists (k = 0.10).

For the category cycling facilities, all items demonstrated fair or poor validity. The lowest score was found for the item regarding path condition and smoothness of the cycle lane (k = -0.03).

In the subscale pedestrian facilities, all items showed moderate-to-perfect agreement, with the highest score found for presence of the sidewalk (k = 0.91)).

For the subscale aesthetics, one item demonstrated poor validity (“presence of trees” (k = 0.10)), the remaining items showed moderate-to-substantial agreement (4 items).

## Discussion

This study evaluated intra-rater reliability, inter-rater reliability and criterion validity of a Google Street View-based audit to virtually assess physical environmental characteristics along cycling routes to school among 11-to-12-year old children. Because in future studies, the audit instrument will also be used to assess environmental characteristics for the entire cycling routes and not just for segments, we opted to analyze the reliability and validity at the level of the entire cycling route. Overall, 78% of all items of EGA-Cycling generated high intra-rater reliability and the inter-rater reliability was acceptable for 43% of all items. Acceptable criterion validity between the ratings by Google Street View and the on-site ratings was reported for 54% of all items.

The reliability results found in the present study are comparable with previous studies [32,52]. Griew and colleagues [52] rated the neighborhood area, including street design factors related to walking among adults, with a newly developed street audit using Google Street View. In line with our intra-rater results, they found high intra-rater reliability scores for all street characteristics. Overall, studies evaluating audit tools to assess the physical environment stressed the difficulty to judge on quality or aesthetics [21,53]. Griew and colleagues [52] found low agreement between raters for pavement quality, lighting and road permeability, also indicating that judgment on quality or aesthetics differed between raters due to subjectivity. The same conclusion was made in a study of Kelly and colleagues [32] when evaluating the inter-rater reliability of the Active Neighborhood Checklist using Google Street View. They found low scores for parking facilities, tree shade, sidewalk width and curb cuts. In our results, divergent scores were also found, ranging from no to almost perfect inter-rater reliability. Low inter-rater reliability scores (found for “openness view”, “presence of driveways”, “presence of garage doors”, “type of cycle lane”, “width of cycle lane”, two-way cycle lane”, “maintenance of cycle lane”, “lighting of cycle lane” and “maintenance of front yards”) could be explained by the subjective interpretation of the observers. A clear definition of those items and their response options was difficult to provide, so ratings between both observers could differ. Therefore, training with specific instructions and examples of different environments to interpret and rate those subjective items has to be provided for different observers. Regarding the type of cycle lane, observers received no clear instructions for scoring this item when different types of cycle lanes occurred in the same street segment. They had to choose one specific type of cycle lane that fitted the best in the street segment, mostly depending on their interpretation. Adapting the response options for this item (e.g. adding “mixed type of cycle lane” or multiple response options) could be a possible solution to increase inter-rater reliability.

Furthermore, little variance in the answers could explain low inter-rater reliability scores for residential mix and swerving alternatives for cyclists, as the percentage agreement for both items was generally high (>70% percentage agreement).

When validating the ratings by Google Street View against the audit filled out by on-site assessments, mixed results were found. Large variations in the criterion validity scores were reported, however with acceptable values for approximately 54% of all items. Of all low-scored items, 4 items (31%) showed high percentage agreement (>70.0%), indicating low variance in the items regarding presence of recreational destinations, streetlights in the street segment, two-way cycle lane and presence of trees. Since high percentage agreement was found for those items, acceptable criterion validity between the different answers can be assumed.

Our results showed somewhat lower criterion validity scores compared to similar studies that conducted virtual audits in a neighborhood area [26,27,30,52]. However, only Badland and colleagues [26] included features specifically related to cycling (e.g. “path type, slope, curb type and condition of cycle lane”, “one-road cycle lane”). In our study, the majority of all low-scored items was reported for cycling facilities compared to the other subscales. All items categorized under cycling facilities, except one (“surface of cycle lane”), had poor or fair validity. In contrast, Badland and colleagues [26] reported a high criterion validity score for the items related to cycling. However, they included all individual items in one category (“cycling surface”) and calculated the agreement for the category and not for the individual items. Additionally, the majority of those similar studies found lowest validity scores for qualitative and detailed features [26,27,29,30], street condition features [27,30], and changeable items like presence of graffiti and litter [26,27,29,30]. In our study, some low-scored validity items (“openness of the view”, “presence of driveways”, “maintenance of cycle lane”, “lighting of cycle lane”) were assessed through a qualitative judgment, so subjective interpretation of the items by the observers could explain those low scores. Additionally, the perspective of the camera when Google Street View images were captured makes it sometimes difficult to observe more detailed features [32]. This could also explain the low scores for the items regarding swerving alternatives for cyclists, width of the cycle lane and path condition of the cycle lane. For example, the path condition of the cycle lane was easier to rate when going on-site and experience it by actually cycling the routes, than rating it through Google Street View.

Another possible explanation for low criterion validity scores (for the items “measures that can slow down traffic”, “type of cycle lane”) could be that the Belgian virtual images in Google Street View dated from 2009, while on-site assessment was conducted in winter 2013. Similar studies did not only highlight the difficulty to audit temporal items (e.g. graffiti, litter,…) [27-30,32,33], but did also report the temporal lag between the Google Street View images and the on-site assessments as a limitation to use Google Street View [27-29,54]. There is no fixed frequency in which new Google Street View images are collected, however, an update of the virtual images appears once every 5 years. The date when images are taken is shown in Google Street View. Additionally, Google Street View provides information on when and where new images will be taken [55]. This enables the possibility to select areas, where the Google Street View images are not out dated and to focus on these areas for certain research purposes. Curtis and colleagues [54] investigated the spatio-temporal stability in the Google Street View dates. They concluded that the dates of the images often changed and without warning. For example, images of Google Street View can be presented for one date and can suddenly change to images from another date. Those changes mostly occur at intersections. So, when using Google Street View as a data collection tool, researchers should be aware of these issues. Additionally, the new history function of Google Street View provides the possibility for the user to travel to the past to see how a place has changed over the years [55]. Google Street View gathered historical imagery from past Street View collections dating back to 2007. This function allows identifying changes in the physical environment, which might be of interest in some studies. According to the Flemish agency for roads and traffic, many infrastructural changes in the Flemish traffic landscape (e.g. construction of new cycle lanes) were conducted after 2009 [56]. So recent changes could not be observed in Google Street View, while on-site ratings showed for those items other and new infrastructural elements (e.g. separated cycle lane, speed bump).

So, actually cycling along the routes and observing by on-site assessment are the preferred method to assess features related to the micro-environment (e.g. cycle lane condition) and new infrastructural features (e.g. separate cycle lanes not allowing car traffic). However, for the other items conducting the audit through Google Street View remains beneficial since there is a large gain in time (including travel and rating time). Traveling to and from the different cycling routes requires more effort and time when observing the environment by on-site assessments compared to observations using Google Street View. An additional added value of Google Street View is the fact that when items are unclear, they can easily be double checked while it requires much more time and effort to go back to the specific location through field observations.

For many items a constant response was recorded and it mostly appeared in the Google Street View ratings [see Additional file 2]. Difficulties to see the presence of some detailed features with Google Street View, for example the maintenance of the street segment, could explain this. This may demonstrate that assessing the physical environment through Google Street View, especially for more detailed and qualitative features, may give less nuanced results. However, two items had a constant response given by all observers (“maintenance buildings” and “presence of graffiti and litter”). For observing the physical environment in substantial regions, it is suggested to rate these items in other regions. Although a study in the Netherlands by de Vries and colleagues [14] found that litter was not associated with cycling to school among elementary school children, removing those items from the audit representing all environments could be premature. The presence of graffiti and litter may nevertheless influence cycling behavior in other regions [57].

The present study has some important limitations. One limitation involved the small number of raters that conducted EGA-Cycling, which may affect the reliability of the results. Secondly, conducting the study only among 6^th^ graders limits generalization to all primary school children. The study also included only one school situated in a suburban area. Third, the cycling route to school from each child was obtained through the parents. However, the actual cycling route that children take to school may differ from what parents consider as the actual route, especially in older and more independent children. Future research could use GPS devices to track in detail the actual routes that children take to school or in leisure time.

The present study has some important strengths. To our knowledge this is one of the first studies that tested both intra- and inter-rater reliability, and added the criterion validity of a newly developed Google Street View-based audit focusing on cycling routes to school. Google Street View provides many advantages to assess the physical environment. It is an objective method, cost and time effective, always available and does not have to take weather conditions into account. The present study can provide direction to research that assesses the physical environment along cycling routes. To assess macro-environmental features along cycling routes to school, EGA-Cycling is a helpful instrument. However, to assess environmental features on a micro-level in a cycling setting (detailed and temporary features specifically related to cycling), on-site assessments should be added to the observations through Google Street View. Furthermore, it is of interest that future research continues to evaluate the use of Google Street View to assess the physical environment across other settings and other populations.

## Conclusions

The Google Street View-based audit (EGA-Cycling) generated acceptable reliability and validity and can be valuable to virtually assess physical environmental characteristics along cycling routes to school among 11-to-12-year old children, demonstrating less resource- and time-intensive work. However, for features observing the micro-environment and specifically related to cycling, on-site assessments should be added to the observations through Google Street View.

## Competing interests

The authors declare they have no competing interests.

## Authors’ contributions

GV conducted the statistical analyses and drafted the manuscript. GV and FD developed EGA-Cycling, the data collection protocol and coordinated the data collection. GC, IDB and DVD participated in the interpretation of the data, helped to draft the manuscript and revised the manuscript for important intellectual content. All authors read and approved the final manuscript.

## Supplementary Material

Additional file 1 **Outline of EGA-Cycling and relevance to children’s cycling behavior to school.** This file provides an outline of EGA-Cycling, respectively the individual items with response options and their relevance to children’s cycling behavior to school. Click here for file

Additional file 2 **Answer frequencies on first assessment of EGA-Cycling.** This file provides the response frequency of each item on the first assessment of the instrument audited by rater 1, rater 2 and by on-site rating. Click here for file

Additional file 3 **Intra-rater reliability, inter-rater reliability and criterion validity of EGA-Cycling.** This file provides the results regarding intra-rater, inter-rater reliability and criterion validity of EGA-Cycling to assess the physical environment along cycling routes to school. Click here for file
