# Supplementary material for: Assessing the environmental characteristics of cycling routes to school: a study on the reliability and validity of a Google Street View-based audit
Source: Int J Health Geogr. 2014 Jun 10;13:19. doi: 10.1186/1476-072X-13-19 (PMC4063420; doi:10.1186/1476-072X-13-19)
Supplement: Additional file 1 — Outline of EGA-Cycling and relevance to children’s cycling behavior to school. This file provides an outline of EGA-Cycling, respectively the individual items with response options and their relevance to children’s cycling behavior to school. [file 1476-072X-13-19-S1.pdf]

Outline of EGA-Cycling and relevance to children's cycling behavior to school

Item	*Response option*	*Relevance for children's cycling behavior*
Land use		
1) Are residential and non-residential land uses visible in this segment?	No/Yes	Item included to assess land use mix diversity. Land use mix diversity has a positive association with walking and cycling to school [35,49].
2) What types of buildings are visible in this segment?	Single buildings/ Closed or semi-detached buildings/Apartment buildings/Not applicable	Item included to assess residential density. Residential density has a positive association with walking and cycling to school [35,50].
3) Are commercial destinations visible in this segment (restaurant, shop, tank station, …)?	No/Yes	Item included to assess accessibility to facilities. The more access to facilities, the more likely children commute actively to school [13].
4) Is heavy industry visible in this segment (industrial sites, …)?	No/Yes	Item included to assess land use mix diversity. Land use mix diversity has a positive association with walking and cycling to school [35,49].
5) Are public destinations visible in this segment (school, police station, bus stop, …)?	No/Yes	Item included to assess accessibility to facilities. The more access to facilities, the more likely children commute actively to school [13].
6) Are recreational destinations visible in this segment (fitness, playground …)?	No/Yes	Item included to assess accessibility to facilities. The more access to recreational facilities, the more likely children commute actively to school [13,14].
7) Are natural features visible in this segment (river, lake, …)?	No/Yes	Items included to assess land use mix diversity. Land use mix diversity has a positive association with walking and cycling to school [35,49].
8) Is this segment characterized by an open or closed view?	Open view/Not open-closed view/Closed view	
Characteristics of the street segment		
A. General characteristics		
1) What is the road type?	One road for one-direction-traffic/One road not divided into lanes/One road divided in one lane each direction / One road divided in two lanes each direction/Two roads divided in one lane each direction/Two roads divided in two lanes each direction	Items included to assess traffic safety (road safety).<br>Positive associations between road safety-related infrastructure (such as traffic lights, pedestrian crossings, access to local roads with lower speed limits and lower traffic volume than main roads) and active transport among school-aged children [51].
2) What is the posted speed limit on this segment?	30 km/h<br>50 km/h<br>70 km/h<br>90 km/h	

3) Are there measures on this segment that can slow down traffic?<br>Mark all that apply<br>- Roundabout<br>- Traffic light<br>- Speed bump<br>- Speed ramp<br>- Traffic slalom<br>- Lane narrowing	No/Yes<br><br><br>No/Yes<br>No/Yes<br>No/Yes<br>No/Yes<br>No/Yes<br>No/Yes	
4) Are there measures on this segment that make it easier for pedestrians/cyclists to cross over?<br>Mark all that apply<br>- Crosswalk<br>- Marked crosswalk for cyclists<br>- Traffic lights<br>- Traffic island<br>- Kerb extension<br>- Underpass for pedestrians or cyclists	No/Yes<br><br><br><br>No/Yes<br>No/Yes<br>No/Yes<br>No/Yes<br>No/Yes<br>No/Yes	
5) Is the street segment well maintained?	No/Yes	
6) Are streetlights present in this street segment?	No/Yes	Item included to assess traffic and crime safety. Lighting is positively associated with children's active commuting [52].
7) What type of vehicle parking facilities is provided in this street segment?	On street/Next to the street (front yard, adjacent piece of land)/On adjacent parking / On separate parking / No parking	Item included to assess traffic safety (traffic danger). Parental concern about dangerous traffic is negatively associated with children's active commuting behavior [13].
8) How steep or hilly is this segment?	Flat/Gentle slope/Moderate slope/Steep slope	Item included to assess steepness. Altitude differences decrease children's cycling behavior [34,53].
9) Are there swerving alternatives for cyclists (front yard, …)?	No/Yes	Item included to assess traffic safety (traffic danger). Parental concern about dangerous traffic is negatively associated with children's active commuting behavior [13].
10) How many buildings have windows on the street side to have sight on cyclists?	No buildings with windows on street side/Few buildings with windows on street side/ Many buildings with windows on street side	Item included to assess crime safety. Positive association between persons having sight on children and active commuting to school [13,34,54].

11) How many buildings have driveways where vehicles suddenly can pop up?	No driveways/Approx. 25% buildings have one driveway/ Approx. 50% buildings have one driveway/Most buildings have one driveway	Items included to assess traffic safety (traffic danger). Parental concern about dangerous traffic is negatively associated with children's active commuting behavior [13].
12) How many buildings have garage doors facing the street?	No garages/Approx. 25% buildings have one garage/ Approx. 50% buildings have one garage/Most buildings have one garage	
Cycling facilities		
1) What type of cycle lane is visible in this segment?	Cycle lane separated from the road /Adjoining cycle lane (slightly increased) / Cycle lane is part of the road (white broken lines) / Cycle lane (non-compulsory or of a different color)/ No cycle lane	Item included to assess cycling facilities. Parental concerns of children's active commuting are related to presence and quality of walking and cycling facilities [35]. Cycle lanes have a positive impact on cycling to school [14].
2) What is the width of the cycle lane?	Small (space for 1 cyclist)/Wide (space for 2 cyclists)/Not applicable	Item included to assess cycling facilities. Small cycle lanes can be a risk for accidents concerning cyclists [38].
3) Is it a two-way cycle lane?	No/Yes/Not applicable	Item included to assess cycling facilities. Item contributes to description of cycle lanes.
4) Is the cycle lane well maintained?	No/Yes/Not applicable	Item included to assess cycling facilities. Parental concerns of children's active commuting are related to presence and quality of walking and cycling facilities [35].
5) Does lighting cover the cycle lane area?	No/Yes/Not applicable	Item included to assess traffic and crime safety. Lighting is positively associated with children's active commuting [52].
6) What is the surface of the cycle lane? (If no cycle lane is present, evaluate the road)	Bitumen/Continuous concrete/Paving bricks/Concrete slabs/Cobblestones/Gravel	Item included to assess cycling facilities. Item contributes to description of cycle lanes.
7) What is the path condition and smoothness?	Poor (a lot of bumps, cracks, holes)/Moderate (some bumps, cracks, holes)/Good (very few bumps, cracks, holes)	Item included to assess cycling facilities. Parental concerns of children's active commuting are related to presence and quality of walking and cycling facilities [35].

Pedestrian facilities		
1) Is there a sidewalk visible in this segment?	No/Yes/Not applicable	Item included to assess pedestrian facilities. Parental concerns of children's active commuting are related to presence and quality of walking and cycling facilities [35]. Sidewalks have a positive association with children's walking and cycling to school [55,56].
2) Is the sidewalk well maintained?	No/Yes/Not applicable	Item included to assess pedestrian facilities. Parental concerns of children's active commuting are related to presence and quality of walking and cycling facilities [35].
3) Does lighting cover the sidewalk area?	No/Yes/Not applicable	Item included to assess traffic and crime safety. Lighting is positively associated with children's active commuting [52].
Aesthetics		
1) Are trees visible in this segment (e.g. avenue of trees)?	No/Yes	Items included to assess the aesthetics. Aesthetics along routes to school have an influence on parental concerns of children's active commuting to school [35], however conflicting results are still reported [57].
2) Are attractive buildings visible in this segment (historical buildings, architectural design, building variety)?	No/Yes	
3) Are the buildings well maintained in this segment?	No/Yes/Not applicable	
4) Are front yards visible in this segment?	No/Yes	
5) Are the front yards well maintained?	No/Yes/Not applicable	
6) Are attractive natural features visible in this segment?	No/Yes	
7) Are graffiti and litter apparent on this segment?	No/Yes	
