# Supplementary material for: Assessing the environmental characteristics of cycling routes to school: a study on the reliability and validity of a Google Street View-based audit
Source: Int J Health Geogr. 2014 Jun 10;13:19. doi: 10.1186/1476-072X-13-19 (PMC4063420; doi:10.1186/1476-072X-13-19)
Supplement: Additional file 2 — Answer frequencies on first assessment of EGA-Cycling. This file provides the response frequency of each item on the first assessment of the instrument audited by rater 1, rater 2 and by on-site rating. [file 1476-072X-13-19-S2.pdf]

Answer frequencies on first assessment of EGA-Cycling

Item	Response option	Rater 1<br>Answer frequency (%)	Rater 2<br>Answer frequency (%)	On-site rating<br>Answer frequency (%)
Land use				
1) Are residential and non-residential land uses visible in this segment?	No	3.3	13.3	20.0
	Yes	96.7	86.7	80.0
2) What types of buildings are visible in this segment?	Single buildings	20.0	20.0	23.3
	Closed or semi-detached buildings	73.3	76.7	73.3
	Apartment buildings	6.7	3.3	0.0
	Not applicable	0.0	0.0	3.3
3) Are commercial destinations visible in this segment (restaurant, shop, tank station, …)?	No	33.3	30.0	40.0
	Yes	66.7	70.0	60.0
4) Is heavy industry visible in this segment (industrial sites, …)?	No	96.7	96.7	96.7
	Yes	3.3	3.3	3.3
5) Are public destinations visible in this segment (school, police station, bus stop, …)?	No	46.7	40.0	40.0
	Yes	53.3	60.0	60.0
6) Are recreational destinations visible in this segment (fitness, playground, …)?	No	93.3	100	90.0
	Yes	6.7	0.0	10.0
7) Are natural features visible in this segment (river, lake, …)?	No	70.0	63.3	70.0
	Yes	30.0	36.7	30.0
8) Is this segment characterized by an open or closed view?	Open view	3.3	3.3	23.3
	Not open-closed view	23.3	60.0	73.3
	Closed view	73.3	36.7	3.3

Item	Response option	Rater 1<br>Answer frequency (%)	Rater 2<br>Answer frequency (%)	On-site rating<br>Answer frequency (%)
Characteristics of the street segment				
A. General characteristics				
1) What is the road type?	One road for one-direction-traffic	10.0	3.3	6.7
	One road not divided into lanes	3.3	6.7	40.0
	One road divided in one lane each direction	86.7	90.0	53.3
	One road divided in two lanes each direction	0.0	0.0	0.0
	Two roads divided in one lane each direction	0.0	0.0	0.0
	Two roads divided in two lanes each direction	0.0	0.0	0.0
2) What is the posted speed limit on this segment?	30 km/h	6.7	13.8	16.7
	50 km/h	76.7	72.4	66.7
	70 km/h	10.0	10.3	13.3
	90 km/h	6.7	3.4	3.3
3) Are there measures on this segment that can slow down traffic?	No	76.7	36.7	43.3
	Yes	23.3	63.3	56.7
Mark all that apply				
- Roundabout	No	100	100	93.3
	Yes	0.0	0.0	6.7
- Traffic light	No	93.3	96.7	96.7
	Yes	6.7	3.3	3.3
- Speed bump	No	86.7	56.7	93.3
	Yes	13.3	43.3	6.7
- Speed ramp	No	96.7	96.7	73.3
	Yes	3.3	3.3	26.7
- Traffic slalom	No	100	100	100
	Yes	0.0	0.0	0.0
- Lane narrowing	No	96.7	63.3	86.7
	Yes	3.3	36.7	13.3

Item	Response option	Rater 1<br>Answer frequency (%)	Rater 2<br>Answer frequency (%)	On-site rating<br>Answer frequency (%)
4) Are there measures on this segment that make it easier for pedestrians/cyclists to cross over?	No	26.7	26.7	26.7
	Yes	73.3	73.3	73.3
Mark all that apply				
- Crosswalk	No	26.7	26.7	26.7
	Yes	73.3	73.3	73.3
- Marked crosswalk for cyclists	No	90.0	93.3	86.7
	Yes	10.0	6.7	13.3
- Traffic lights	No	90.0	96.7	96.7
	Yes	10.0	3.3	3.3
- Traffic island	No	100	100	96.7
	Yes	0.0	0.0	3.3
- Kerb extension	No	96.7	73.3	86.7
	Yes	3.3	26.7	13.3
- Underpass for pedestrians or cyclists	No	100	100	100
	Yes	0.0	0.0	0.0
5) Is the street segment well maintained?	No	0.0	0.0	10.0
	Yes	100	100	90.0
6) Are streetlights present in this street segment?	No	6.7	0.0	0.0
	Yes	93.3	100	100
7) What type of vehicle parking facilities is provided in this street segment?	On street	60.0	46.7	33.3
	Next to the street (front yard, adjacent piece of land)	13.3	10.0	10.0
	On adjacent parking	26.7	33.3	43.3
	On separate parking	0.0	3.3	0.0
	No parking	0.0	6.7	13.3

Item	Response option	Rater 1	Rater 2	On-site rating
		Answer frequency (%)	Answer frequency (%)	Answer frequency (%)
8) How steep or hilly is this segment?	Flat	100	100	90.0
	Gentle slope	0.0	0.0	10.0
	Moderate slope	0.0	0.0	0.0
	Steep slope	0.0	0.0	0.0
9) Are there swerving alternatives for cyclists (front yard, …)?	No	3.3	3.3	60.0
	Yes	96.7	96.7	40.0
10) How many buildings have windows on the street side to have sight on cyclists?	No buildings with windows on street side	0.0	0.0	3.3
	Few buildings with windows on street side	40.0	43.3	46.7
	Many buildings with windows on street side	60.0	56.7	50.0
11) How many buildings have driveways where vehicles suddenly can pop up?	No driveways	36.7	10.0	30.0
	Approx. 25% buildings have one driveway	16.7	20.0	13.3
	Approx. 50% buildings have one driveway	10.0	20.0	16.7
	Most buildings have one driveway	36.7	50.0	40.0
12) How many buildings have garage doors facing the street?	No garages	20.0	40.0	33.3
	Approx. 25% buildings have one garage	26.7	23.3	16.7
	Approx. 50% buildings have one garage	20.0	33.3	33.3
	Most buildings have one garage	33.3	3.3	16.7

Item	Response option	Rater 1<br>Answer frequency (%)	Rater 2<br>Answer frequency (%)	On-site rating<br>Answer frequency (%)
B. Cycling facilities				
1) What type of cycle lane is visible in this segment?	Cycle lane separated from the road	36.7	6.7	13.3
	Adjoining cycle lane (slightly increased)	0.0	0.0	0.0
	Cycle lane is part of the road (white broken lines)	13.3	13.3	10.0
	Cycle lane (non-compulsory or of a different color)	0.0	0.0	0.0
	No cycle lane	50.0	80.0	76.7
2) What is the width of the cycle lane?	Small (space for 1 cyclist)	0.0	3.3	6.0
	Wide (space for 2 cyclists)	50.0	16.7	10.0
	Not applicable	50.0	80.0	84.0
3) Is it a two-way cycle lane?	No	6.7	6.7	4.0
	Yes	43.3	13.3	12.0
	Not applicable	50.0	80.0	84.0
4) Is the cycle lane well maintained?	No	0.0	0.0	4.0
	Yes	50.0	20.0	12.0
	Not applicable	50.0	80.0	84.0
5) Does lighting cover the cycle lane area?	No	0.0	0.0	4.0
	Yes	50.0	20.0	12.0
	Not applicable	50.0	80.0	84.0
6) What is the surface of the cycle lane? (If no cycle lane is present, evaluate the road)	Bitumen	40.0	70.0	53.3
	Continuous concrete	0.0	0.0	0.0
	Paving bricks	30.0	6.7	3.3
	Concrete slabs	30.0	23.3	36.7
	Cobblestones	0.0	0.0	0.0
	Gravel	0.0	0.0	6.7
7) What is the path condition and smoothness?	Poor (a lot of bumps, cracks, holes)	0.0	0.0	10.0
	Moderate (some bumps, cracks, holes)	0.0	0.0	16.7
	Good (very few bumps, cracks, holes)	100	100	73.3

Item	Response option	Rater 1<br>Answer frequency (%)	Rater 2<br>Answer frequency (%)	On-site rating<br>Answer frequency (%)
C. Pedestrian facilities				
1) Is there a sidewalk visible in this segment?	No	13.3	13.3	14.0
	Yes	86.7	86.7	86.0
	Not applicable	0.0	0.0	0.0
2) Is the sidewalk well maintained?	No	0.0	0.0	10.0
	Yes	86.7	86.7	76.0
	Not applicable	13.3	13.3	14.0
3) Does lighting cover the sidewalk area?	No	3.3	0.0	0.0
	Yes	83.3	86.7	86.0
	Not applicable	13.3	13.3	14.0
Aesthetics				
1) Are trees visible in this segment (e.g. avenue of trees)?	No	90.0	76.7	86.7
	Yes	10.0	23.3	13.3
2) Are attractive buildings visible in this segment (historical buildings, architectural design, building variety)?	No	96.7	100	93.3
	Yes	3.3	0.0	6.7
3) Are the buildings well maintained in this segment?	No	0.0	0.0	0.0
	Yes	100	100	100
	Not applicable	0.0	0.0	0.0
4) Are front yards visible in this segment?	No	43.3	10.0	43.3
	Yes	56.7	90.0	56.7
5) Are the front yards well maintained?	No	0.0	0.0	3.3
	Yes	56.7	90.0	53.3
	Not applicable	43.3	10.0	43.3

		Rater 1	Rater 2	On-site rating
Item	Response option	Answer frequency (%)	Answer frequency (%)	Answer frequency (%)
6) Are attractive natural features visible in this segment?	No	90.0	70.0	76.7
	Yes	10.0	30.0	23.3
7) Are graffiti and litter apparent on this segment?	No	100	100	100
	Yes	0.0	0.0	0.0
