# Supplementary material for: Assessing the environmental characteristics of cycling routes to school: a study on the reliability and validity of a Google Street View-based audit
Source: Int J Health Geogr. 2014 Jun 10;13:19. doi: 10.1186/1476-072X-13-19 (PMC4063420; doi:10.1186/1476-072X-13-19)
Supplement: Additional file 3 — Intra-rater reliability, inter-rater reliability and criterion validity of EGA-Cycling. This file provides the results regarding intra-rater, inter-rater reliability and criterion validity of EGA-Cycling to assess the physical environment along cycling routes to school. [file 1476-072X-13-19-S3.pdf]

Intra-, inter-rater reliability and criterion validity scores of EGA-Cycling

Item	Response options	Intra-rater reliability		Inter-rater reliability		Criterion validity	
		Kappa	% agreement	Kappa	% agreement	Kappa	% agreement
Land use							
1) Are residential and non-residential land uses visible in this segment?	No/Yes	0.474	93.3	0.366	90.0	0.502	90.0
2) What types of buildings are visible in this segment?	Single buildings/ Closed or semi-detached buildings/Apartment buildings/Not applicable	0.916	96.7	0.579	83.3	0.774	90.0
3) Are commercial destinations visible in this segment (restaurant, shop, tank station, …)?	No/Yes	1.000	100	0.923	96.7	0.595	80.0
4) Is heavy industry visible in this segment (industrial sites, …)?	No/Yes	1.000	100	1.000	100	1.000	100
5) Are public destinations visible in this segment (school, police station, bus stop, …)?	No/Yes	1.000	100	0.865	93.3	0.880	94.0
6) Are recreational destinations visible in this segment (fitness, playground, …)?	No/Yes	0.651	96.7	N/A[a]	93.3	-0.056	88.0
7) Are natural features visible in this segment (river, lake, …)?	No/Yes	1.000	100	0.701	86.7	0.582	80.0
8) Is this segment characterized by an open or closed view?	Open view/Not open-closed view/Closed view	0.915	96.7	0.158	50.0	0.000	30.0

Item	Response options	Intra-rater reliability		Inter-rater reliability		Criterion validity	
		Kappa	% agreement	Kappa	% agreement	Kappa	% agreement
Characteristics of the street segment							
A. General characteristics							
1) What is the road type?	One road for one-direction-traffic/One road not divided into lanes/One road divided in one lane each direction / One road divided in two lanes each direction/Two roads divided in one lane each direction/Two roads divided in two lanes each direction	1.000	100	0.534	90.0	0.553	72.0
2) What is the posted speed limit on this segment?	30 km/h<br>50 km/h<br>70 km/h<br>90 km/h	1.000	100	0.829	90.0	0.667	86.0
3) Are there measures on this segment that can slow down traffic?	No/Yes	0.902	96.7	0.183	53.3	0.385	68.0
Mark all that apply							
- Roundabout	No/Yes	N/A[a]	100	N/A[a]	100	N/A[a]	96.0
- Traffic light	No/Yes	0.651	96.7	0.651	96.7	0.485	96.0
- Speed bump	No/Yes	1.000	100	0.335	70.0	0.179	70.0
- Speed ramp	No/Yes	1.000	100	1.000	100	0.091	72.0
- Traffic slalom	No/Yes	N/A[a]	100	N/A[a]	100	N/A[a]	100
- Lane narrowing	No/Yes	1.000	100	0.112	66.7	0.016	76.0

Item	Response options	Intra-rater reliability		Inter-rater reliability		Criterion validity	
		Kappa	% agreement	Kappa	% agreement	Kappa	% agreement
4) Are there measures on this segment that make it easier for pedestrians/cyclists to cross over?	No/Yes	0.918	96.7	0.830	93.3	0.802	92.0
Mark all that apply							
- Crosswalk	No/Yes	0.918	96.7	0.830	93.3	0.802	92.0
- Marked crosswalk for cyclists	No/Yes	1.000	100	0.783	96.7	0.847	98.0
- Traffic lights	No/Yes	0.474	93.3	0.474	93.3	0.790	98.0
- Traffic island	No/Yes	N/A[a]	100	N/A[a]	100	N/A[a]	98.0
- Kerb extension	No/Yes	1.000	100	0.173	76.7	0.558	92.0
- Underpass for pedestrians or cyclists	No/Yes	N/A[a]	100	N/A[a]	100	N/A[a]	100
5) Is the street segment well maintained?	No/Yes	N/A[a]	96.7	N/A[a]	100	N/A[a]	90.0
6) Are streetlights present in this street segment?	No/Yes	1.000	100	N/A[a]	93.3	-0.027	94.0
7) What type of vehicle parking facilities is provided in this street segment?	On street/Next to the street (front yard, adjacent piece of land)/On adjacent parking / On separate parking / No parking	0.938	96.7	0.460	66.7	0.453	72.0
8) How steep or hilly is this segment?	Flat/Gentle slope/Moderate slope/Steep slope	N/A[a]	100	N/A[a]	100	N/A[a]	92.0

Item	Response options	Intra-rater reliability		Inter-rater reliability		Criterion validity	
		Kappa	% agreement	Kappa	% agreement	Kappa	% agreement
9) Are there swerving alternatives for cyclists (front yard, …)?	No/Yes	1.000	100	-0.034	93.3	0.096	50.0
10) How many buildings have windows on the street side to have sight on cyclists?	No buildings with windows on street side/Few buildings with windows on street side/ Many buildings with windows on street side	0.571	80.0	0.521	76.7	0.455	72.0
11) How many buildings have driveways where vehicles suddenly can pop up?	No driveways/Approx. 25% buildings have one driveway/ Approx. 50% buildings have one driveway/Most buildings have one driveway	0.951	96.7	0.174	40.0	0.302	50.0
12) How many buildings have garage doors facing the street?	No garages/Approx. 25% buildings have one garage/ Approx. 50% buildings have one garage/Most buildings have one garage	0.819	86.7	0.188	36.7	0.453	60.0

		Intra-rater reliability		Inter-rater reliability		Criterion validity	
Item	Response options	Kappa	% agreement	Kappa	% agreement	Kappa	% agreement
B. Cycling facilities							
1) What type of cycle lane is visible in this segment?	Cycle lane separated from the road /Adjoining cycle lane (slightly increased) / Cycle lane is part of the road (white broken lines) / Cycle lane (non-compulsory or of a different color)/ No cycle lane	1.000	100	0.343	63.3	0.333	60.0
2) What is the width of the cycle lane?	Small (space for 1 cyclist)/Wide (space for 2 cyclists)/Not applicable	0.931	96.6	0.226	60.0	0.241	69.4
3) Is it a two-way cycle lane?	No/Yes/Not applicable	0.939	96.7	0.318	63.3	0.368	74.0
4) Is the cycle lane well maintained?	No/Yes/Not applicable	0.933	96.7	0.267	63.3	0.259	70.0
5) Does lighting cover the cycle lane area?	No/Yes/Not applicable	0.933	96.7	0.267	63.3	0.262	70.0
6) What is the surface of the cycle lane? (If no cycle lane is present, evaluate the road)	Bitumen/Continuous concrete/Paving bricks/Concrete slabs/Cobblestones/Gravel	1.000	100	0.465	80.0	0.469	74.0

Item	Response options	Intra-rater reliability		Inter-rater reliability		Criterion validity	
		Kappa	% agreement	Kappa	% agreement	Kappa	% agreement
7) What is the path condition and smoothness?	Poor (a lot of bumps, cracks, holes)/Moderate (some bumps, cracks, holes)/Good (very few bumps, cracks, holes)	N/A[a]	100	N/A[a]	100	-0.030	70.0
C. Pedestrian facilities							
1) Is there a sidewalk visible in this segment?	No/Yes/Not applicable	1.000	100	1.000	100	0.912	98.0
2) Is the sidewalk well maintained?	No/Yes/Not applicable	1.000	100	1.000	100	0.573	86.0
3) Does lighting cover the sidewalk area?	No/Yes/Not applicable	1.000	100	0.872	96.7	0.770	94.0
Aesthetics							
1) Are trees visible in this segment (e.g. avenue of trees)?	No/Yes	1.000	100	0.535	86.7	0.095	74.0
2) Are attractive buildings visible in this segment (historical buildings, architectural design, building variety)?	No/Yes	N/A[a]	96.7	N/A[a]	96.7	0.658	98.0
3) Are the buildings well maintained in this segment?	No/Yes/Not applicable	N/A[a]	100	N/A[a]	100	N/A[a]	100
4) Are front yards visible in this segment?	No/Yes	1.000	100	0.254	66.7	0.625	84.0

Item	Response options	Intra-rater reliability		Inter-rater reliability		Criterion validity	
		Kappa	% agreement	Kappa	% agreement	Kappa	% agreement
5) Are the front yards well maintained?	No/Yes/Not applicable	0.935	96.7	0.254	66.7	0.602	82.0
6) Are attractive natural features visible in this segment?	No/Yes	1.000	100	0.412	80.0	0.419	80.0
7) Are graffiti and litter apparent on this segment?	No/Yes	N/A[a]	100	N/A[a]	100	N/A[a]	100

[a] Unable to be calculated as at least one variable is constant
